# Environmental stress and stallion fertility: mechanistic insights, evidence gaps, and functional implications

**DOI:** 10.3389/fvets.2026.1873966

**Published:** 2026-07-23

**Authors:** Raimonda Tamulionytė–Skėrė, Renata Baltaduonytė, Alius Pockevičius, Urtė Pelenė, Birutė Šlyžienė, Giedrius Palubinskas

**Affiliations:** 1 Large Animal Clinic, Veterinary Academy, Lithuanian University of Health Sciences, Kaunas, Lithuania; 2 Department of Animal Breeding, Faculty of Animal Sciences, Lithuanian University of Health Sciences, Kaunas, Lithuania; 3 Pathology Center, Department of Veterinary Pathobiology, Veterinary Academy, Lithuanian University of Health Sciences, Kaunas, Lithuania; 4 Department of Animal Breeding and Reproduction, Animal Science Institute, Lithuanian University of Health Sciences, Baisogala, Lithuania

**Keywords:** environmental stress, heat stress, oxidative stress, reproductive biomarkers, sperm function, spermatogenesis, stallion fertility

## Abstract

Environmental stress is increasingly recognized as an important determinant of reproductive performance in breeding stallions, particularly in the context of rising temperatures, climate variability, and increasingly intensive management systems. Stallions are especially susceptible because spermatogenesis is highly sensitive to temperature and reproductive activity is regulated by seasonal physiological changes. This review critically evaluates the current understanding of the molecular and cellular mechanisms through which environmental stress affects stallion fertility, with particular emphasis on heat stress, oxidative imbalance, endocrine dysregulation, and sperm functional competence. The available evidence indicates that oxidative stress is currently supported by the strongest stallion-specific data linking environmental stress with impaired reproductive function. In contrast, apoptosis, mitochondrial dysfunction, epigenetic regulation, and fertilization-related signaling pathways are supported primarily by mechanistic studies in other mammalian species and remain insufficiently validated under equine-specific conditions. Environmental stress may compromise not only spermatogenesis but also sperm functional competence, including processes required for successful fertilization, potentially resulting in subfertility despite apparently normal conventional semen parameters. Although mechanistic understanding has advanced considerably, important evidence gaps remain because stallion-specific studies are limited and many proposed mechanisms rely on carefully interpreted cross-species evidence. At present, management-based strategies, including optimization of thermal conditions, housing, and breeding management, provide the strongest evidence for mitigating the adverse effects of environmental stress in stallions. By comparison, antioxidant supplementation, nutritional interventions, molecular biomarkers, and other targeted approaches remain promising but require further validation under equine-specific conditions. Future progress will depend on well-designed stallion-specific studies integrating molecular biomarkers, endocrine profiling, thermophysiological assessment, and comprehensive evaluation of sperm functional competence. Such studies will be essential for improving early detection of reproductive dysfunction and for developing evidence-based strategies to maintain stallion fertility under increasingly challenging environmental conditions.

## Graphical abstract

**Figure:**
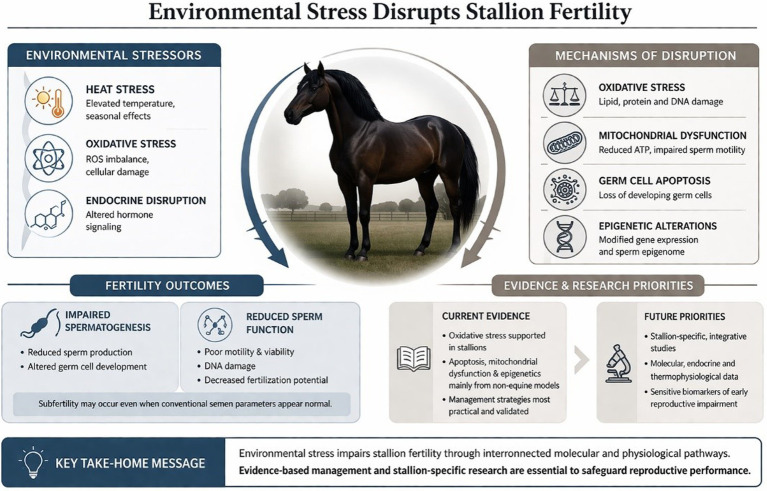

## 1 Introduction

Environmental stress is increasingly recognized as a key determinant of reproductive performance in breeding stallions, particularly in the context of rising global temperatures, climate variability, and the intensification of equine management systems (1, 2). Among environmental stressors, heat stress is considered one of the most important because spermatogenesis is highly temperature-sensitive and requires testicular temperatures to be maintained approximately 2–6 °C below core body temperature for normal function (3, 4). Disruption of this thermoregulatory balance may impair spermatogenesis, reduce semen quality, and compromise fertility, with effects that can extend beyond a single breeding season (5, 6).

In equine breeding systems, reproductive efficiency has substantial economic and genetic importance because of the high individual value of stallions and the central role of selective breeding in maintaining desirable performance and phenotypic traits (7). Despite this, stallion fertility has historically received less focused attention than female reproductive performance, and semen quality is not always systematically prioritized in breeding management. Emerging evidence indicates that environmental stressors—including heat stress, nutritional imbalances, psychological stress, and exposure to environmental pollutants—may interact in complex and potentially synergistic ways to impair male reproductive function.

At the mechanistic level, environmental stress may disrupt several biological systems involved in male reproduction, including the hypothalamic–pituitary–gonadal axis, redox homeostasis, mitochondrial function, germ cell survival, and epigenetic regulation (8–10). Collectively, these effects may compromise not only sperm output but also sperm functional competence, thereby reducing fertilization potential and potentially influencing early embryo development (11).

However, a major limitation in this field is that equine-specific evidence remains fragmented and largely descriptive. Although the mechanisms of stress-induced reproductive dysfunction have been extensively investigated in rodents, livestock, and humans, direct experimental and longitudinal studies in stallions remain limited (6, 12). As a result, much of the current mechanistic framework is extrapolated from other species, which restricts the development of evidence-based, stallion-specific reproductive management strategies.

This limitation is particularly important because the stallion represents a distinct biological and management model. Stallions are characterized by seasonal reproductive activity, species-specific thermoregulatory physiology, intensive breeding use, and housing practices that may increase exposure to both thermal and non-thermal stressors (7, 13). These factors create patterns of reproductive vulnerability that cannot be fully inferred from other domestic male species. In the context of accelerating climate change, more frequent heat waves, and the growing economic significance of equine breeding, understanding the reproductive consequences of environmental stress in stallions is becoming increasingly important.

Therefore, the aim of this review is to provide an integrative, stallion-focused synthesis of how environmental stress affects reproductive function, linking molecular and cellular mechanisms with systemic physiological responses, consequences for spermatogenesis and semen quality, and the practical relevance of available mitigation strategies. By combining mechanistic and applied perspectives, this review aims to identify key knowledge gaps and define priorities for future research needed to support evidence-based reproductive management in breeding stallions under changing environmental conditions.

**Figure 1:**
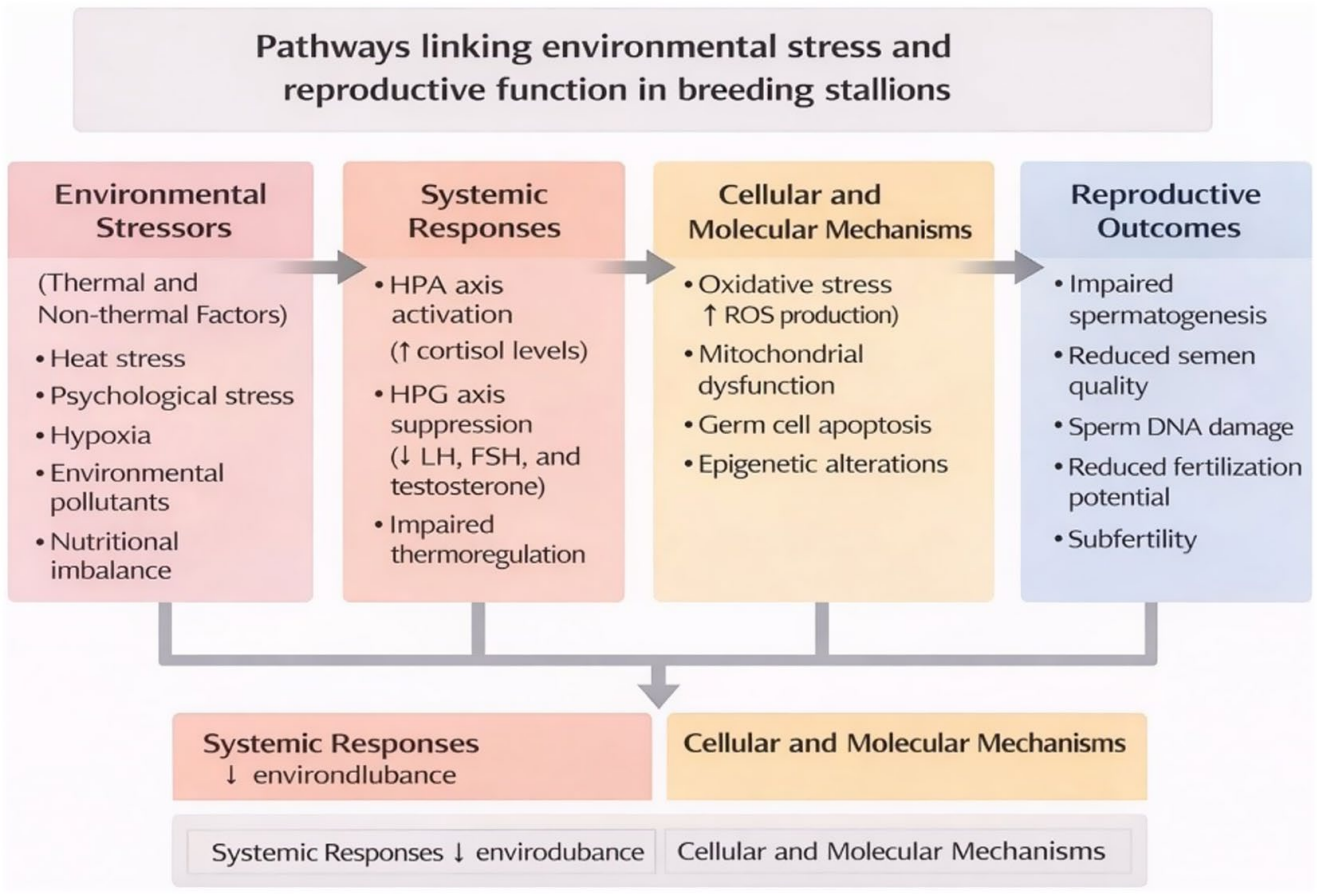
Mechanistic pathways linking environmental stressors to impaired reproductive function in breeding stallions.

To facilitate conceptual understanding of these complex interactions, Figure 1 presents an integrative framework linking environmental stressors with systemic responses, molecular mechanisms, and reproductive outcomes in stallions.

Environmental stressors, including both thermal (heat stress) and non-thermal factors (nutritional imbalances, psychological stress, hypoxia, and environmental pollutants), activate systemic neuroendocrine and thermoregulatory responses that disrupt testicular homeostasis. These responses induce key molecular and cellular mechanisms, including oxidative stress, mitochondrial dysfunction, germ cell apoptosis, and epigenetic alterations. Collectively, these processes impair spermatogenesis, reduce sperm functional competence, and ultimately compromise fertility.

The figure highlights the integrative nature of stress-induced reproductive dysfunction and illustrates how systemic physiological responses and cellular mechanisms interact to influence reproductive outcomes in stallions.

Importantly, this framework should be interpreted as a conceptual synthesis of the currently available evidence rather than as a representation of the relative importance or hierarchy of individual biological mechanisms. While oxidative stress is supported by moderate stallion-specific evidence, several other pathways are inferred primarily from studies in other mammalian species and require validation under equine-specific conditions. Accordingly, the framework is intended to support hypothesis generation, study design, and the identification of research priorities rather than to define dominant mechanisms or prescribe specific management interventions.

## 2 Literature search strategy and selection criteria

The literature included in this review was identified through structured searches of the PubMed, Web of Science, and Scopus databases. Searches were performed using combinations of keywords related to *stallion fertility*, *equine reproduction*, *environmental stress*, *heat stress*, *oxidative stress*, *spermatogenesis*, *sperm quality*, *male fertility*, *endocrine disruption*, *mitochondrial dysfunction*, *epigenetics*, and *reproductive biomarkers*. Boolean operators (AND, OR) were used to refine the search strategy and improve retrieval of relevant publications.

Priority was given to peer-reviewed articles published within the last 10–15 years to ensure that the review reflected current knowledge. Earlier publications were included when they represented landmark studies or provided essential background on reproductive physiology, thermoregulation, oxidative stress, or other biological mechanisms relevant to stallion fertility.

Studies were selected if they addressed the effects of environmental stress on male reproductive function, investigated molecular, cellular, endocrine, or physiological mechanisms associated with reproductive impairment, evaluated semen quality or sperm function, or examined management strategies aimed at reducing stress-related reproductive dysfunction. Original research articles were prioritized, while systematic reviews, meta-analyses, and high-quality narrative reviews were used to support broader biological interpretation and provide additional mechanistic context.

Publications focusing exclusively on female reproduction, studies unrelated to environmental or physiological stress, conference abstracts, editorials, and non-peer-reviewed sources were not considered. Studies that did not contribute relevant information on reproductive mechanisms or fertility outcomes were also excluded.

Because mechanistic studies conducted specifically in stallions remain limited, evidence from other mammalian species, including livestock, rodents, and humans, was incorporated where it provided biologically relevant insights into the mechanisms of stress-induced reproductive dysfunction. Throughout the review, we sought to distinguish evidence supported directly by studies in stallions from findings extrapolated from other species, allowing readers to differentiate between established knowledge and biologically plausible mechanisms requiring further investigation.

The aim of this review was not to perform a formal systematic review or meta-analysis, but rather to provide a critical synthesis of the available evidence, identify current knowledge gaps, and highlight priorities for future research on environmental stress and stallion fertility.

## 3 Molecular and cellular mechanisms of stress-induced reproductive dysfunction

Environmental stress, particularly heat stress, impairs male reproductive function through a network of interconnected molecular and cellular pathways. These include oxidative stress, activation of germ cell death pathways, mitochondrial dysfunction, and epigenetic alterations, which collectively disrupt spermatogenesis and sperm function (6, 10).

Although these mechanisms are well characterized in mammalian models, their specific roles, relative contributions, and interactions in stallions remain incompletely defined. Consequently, current mechanistic understanding relies partly on biologically plausible extrapolation from other species, highlighting the need for critical evaluation of the available evidence.

Accordingly, the following sections distinguish, whenever possible, between mechanisms supported by stallion-specific evidence and those that are currently inferred primarily from studies in other mammalian species.

### 3.1 Oxidative stress and reactive oxygen species (ROS)

Oxidative stress is considered a central mechanism linking environmental stress to impaired male fertility. It occurs when the production of reactive oxygen species (ROS) exceeds antioxidant defense capacity, leading to lipid peroxidation, DNA fragmentation, and protein oxidation (10).

In spermatozoa, excessive ROS compromises membrane integrity, reduces motility, and impairs fertilization potential (11). Stallion sperm cells are particularly vulnerable due to their high content of polyunsaturated fatty acids and limited cytoplasmic antioxidant protection (7). Heat stress further exacerbates this imbalance by increasing ROS production and weakening antioxidant defenses.

In stallions, evidence for oxidative stress is moderate and is primarily based on associations between oxidative markers, seasonal variation, and semen quality. In contrast, strong experimental evidence from rodents, livestock, and humans demonstrates causal links between ROS overproduction and impaired spermatogenesis.

Therefore, oxidative stress represents the most consistently supported and biologically plausible mechanism in stallions, although detailed molecular pathways remain incompletely characterized.

### 3.2 Apoptosis and germ cell loss

Apoptosis plays a key role in stress-induced disruption of spermatogenesis. Elevated testicular temperature and other stressors activate programmed cell death pathways, particularly affecting spermatocytes and spermatids. This process is associated with increased expression of pro-apoptotic factors such as BAX and reduced expression of anti-apoptotic proteins such as BCL-2 (14).

Heat stress may also disrupt the progression of germ cell development, altering the balance between proliferation and degeneration within the seminiferous epithelium (3). Sublethal stress does not always result in complete cell loss but may lead to the production of morphologically abnormal or functionally compromised spermatozoa.

In stallions, evidence for apoptosis is limited and is largely based on histological observations and reductions in semen quality. In contrast, strong mechanistic evidence from experimental models clearly demonstrates heat-induced activation of apoptotic pathways.

Thus, apoptosis represents a highly plausible mechanism in stallions, but its molecular regulation and quantitative contribution to fertility decline remain insufficiently defined.

### 3.3 Mitochondrial dysfunction and energy metabolism

Mitochondria are essential for sperm function, as they regulate ATP production, redox balance, and apoptotic signaling. Proper mitochondrial activity is required to sustain sperm motility and fertilization capacity (8).

Environmental stress disrupts mitochondrial function by reducing membrane potential, impairing ATP generation, and altering mitochondrial dynamics. Heat stress has been shown to induce excessive mitochondrial fission and increase ROS production, creating a self-amplifying cycle of oxidative damage (15).

In stallions, mitochondrial involvement is supported mainly by indirect evidence, particularly through observed reductions in sperm motility and alterations in midpiece integrity. However, detailed mechanistic insights are largely derived from rodent and livestock models, where mitochondrial dysfunction is well established.

Therefore, mitochondrial dysfunction is strongly supported as a mechanistic pathway but remains insufficiently validated in stallion-specific studies.

### 3.4 Epigenetic alterations and transgenerational effects

Environmental stress may induce epigenetic modifications in the male germline, including changes in DNA methylation, histone modification, and non-coding RNA regulation. These processes are essential for normal spermatogenesis and sperm maturation (9). Oxidative stress can disrupt chromatin structure and gene expression, leading to impaired sperm function. Additionally, alterations in small non-coding RNAs, such as PIWI-interacting RNAs (piRNAs), have been associated with genomic instability and impaired germ cell integrity (14).

In addition to piRNAs, microRNAs (miRNAs) represent a key class of small non-coding RNAs involved in the post-transcriptional regulation of gene expression. miRNAs play an important role in spermatogenesis, sperm maturation, motility, and fertilization competence, and they have also been implicated in early embryo development through the delivery of regulatory RNA cargo by spermatozoa (16, 17). Evidence from human and animal studies indicates that environmental stressors, particularly heat stress and oxidative stress, can alter sperm miRNA profiles, potentially affecting both sperm function and embryo developmental potential (9, 11, 17). However, the role of miRNAs in stallion sperm under environmental stress conditions has not yet been systematically investigated.

Importantly, epigenetic changes may have transgenerational consequences, potentially affecting offspring development and reproductive capacity. This broader biological relevance makes epigenetic regulation particularly important in the context of breeding stallions, where reproductive performance has both immediate and long-term genetic implications.

In stallions, direct evidence for epigenetic alterations remains very limited, with no comprehensive molecular studies currently available. In contrast, moderate to strong evidence from human and rodent studies supports a significant role for epigenetic dysregulation in male infertility and impaired offspring outcomes (9, 11).

Thus, epigenetic mechanisms represent a promising but still largely hypothetical pathway in stallion reproductive biology and a major priority for future research.

To provide a structured comparison of the principal molecular mechanisms involved in stress-induced reproductive dysfunction, and to distinguish between stallion-specific evidence and cross-species support, the major pathways and their relative evidentiary strength are summarized in Table 1.

**Table 1:** Molecular mechanisms of environmental stress-induced reproductive dysfunction: comparison of stallion-specific evidence and evidence from other mammalian species.

Mechanism	Principal biological processes	Evidence in stallions	Evidence in other mammalian species	Overall level of evidence	Functional implications for fertility	Key references
Oxidative stress	Excessive ROS production, lipid peroxidation, DNA fragmentation, protein oxidation	Moderate evidence (associations with semen quality, oxidative biomarkers, seasonal variation)	Strong evidence (rodents, livestock, humans)	High	Reduced sperm motility, increased DNA damage, impaired fertilization potential	(10, 11)
Apoptosis	Activation of BAX/BCL-2 pathways, germ cell loss, disruption of spermatogenesis	Limited evidence (histological observations and reduced semen quality)	Strong evidence (experimental rodent and livestock models)	Moderate	Reduced sperm production, abnormal spermatogenesis	(3, 14)
Mitochondrial dysfunction	Reduced ATP production, mitochondrial depolarization, ROS amplification	Limited evidence (indirect associations with sperm motility and midpiece abnormalities)	Strong evidence (rodents and livestock)	Moderate	Impaired sperm motility, altered energy metabolism, increased oxidative damage	(8, 15)
Epigenetic alterations	DNA methylation, histone modification, dysregulation of miRNAs/piRNAs	Very limited evidence (no comprehensive molecular studies in stallions)	Moderate to strong evidence (rodents, humans)	Low	Impaired sperm function, altered embryo development, potential transgenerational effects	(9, 17)
Sperm functional signaling (PLCζ and related pathways)	Oocyte activation, calcium oscillations, fertilization signaling	No direct evidence	Moderate evidence (humans and laboratory animals)	Low	Reduced fertilization competence despite normal semen parameters	(11, 18)

Comparison of the principal molecular mechanisms (Table 1.) implicated in environmental stress-induced reproductive dysfunction, highlighting differences between evidence obtained directly in stallions and mechanistic evidence derived from other mammalian species. Overall level of evidence represents a qualitative assessment based on the quantity, consistency, and biological relevance of the available literature and should not be interpreted as a formal evidence-grading system.

Summary of the principal molecular pathways involved in stress-induced reproductive impairment, highlighting differences in the strength of evidence between stallions and other species. While several mechanisms are biologically well supported, most mechanistic insights are derived from non-equine models.

### 3.5 Sperm functional competence and fertilization-related signaling

Environmental stress may impair not only sperm production and morphology but also sperm functional competence, which is essential for successful fertilization. Functional competence encompasses processes such as capacitation, membrane remodeling, acrosome reaction, and sperm–oocyte interaction, all of which depend on tightly regulated biochemical and signaling pathways. These processes are highly sensitive to oxidative stress, membrane lipid peroxidation, and mitochondrial dysfunction, which may compromise fertilization capacity even when conventional semen parameters appear within normal ranges (11).

A critical component of sperm functional competence is the ability to induce oocyte activation after fertilization. This process is mediated by sperm-borne oocyte-activating factors, among which phospholipase C zeta (PLCζ) plays a central role. PLCζ is responsible for triggering intracellular calcium oscillations in the oocyte, which are essential for the initiation of embryonic development. Disruption of PLCζ expression, localization, or activity has been associated with fertilization failure and early embryonic arrest in several mammalian species (18).

Environmental stress, particularly heat stress and oxidative stress, may interfere with sperm intracellular signaling pathways and protein integrity, potentially affecting PLCζ function. Oxidative damage to sperm proteins and membranes may alter the delivery or activity of oocyte-activating factors, thereby reducing fertilization efficiency. Although this mechanism has been demonstrated primarily in human and laboratory animal studies, it provides a biologically plausible link between environmental stress and reduced fertilization success.

In stallions, direct evidence for stress-induced impairment of sperm functional competence at the molecular signaling level, including PLCζ-related pathways, remains very limited, with no comprehensive studies currently available. In contrast, moderate to strong evidence from human and experimental animal models supports a critical role of PLCζ in fertilization and highlights its sensitivity to cellular stress and sperm quality defects (11, 18).

Thus, impairment of sperm functional competence, including potential disruption of PLCζ-mediated oocyte activation, represents a biologically plausible but largely unexplored mechanism contributing to reduced fertility in stallions under environmental stress conditions. This pathway may be particularly important in explaining cases of subfertility where standard semen quality parameters do not fully reflect fertilization potential.

### 3.6 Integrative perspective and evidence gaps

Current understanding of stress-induced reproductive dysfunction in stallions remains constrained by the limited availability of species-specific mechanistic studies. Consequently, much of the biological framework presented in this review is derived from investigations in other mammalian species, where cellular and molecular responses to environmental stress have been examined in considerably greater detail. Although these findings cannot be directly extrapolated to stallions, they provide valuable insight into conserved biological pathways and identify mechanisms that warrant validation under equine-specific conditions. Throughout this review, evidence from non-equine species is therefore used to support biological interpretation and hypothesis generation rather than as direct confirmation of stallion-specific mechanisms.

Comparison of the available evidence reveals a clear discrepancy between mechanistic plausibility and species-specific validation. Among the mechanisms reviewed, oxidative stress is supported by the strongest body of stallion-specific evidence, whereas apoptosis, mitochondrial dysfunction, epigenetic alterations, and sperm functional signaling remain supported largely by indirect observations or by studies conducted in other mammalian species.

Even for oxidative stress, however, the available evidence should be interpreted with caution. Most studies are observational, include relatively small numbers of animals, and differ considerably in breed composition, environmental conditions, semen evaluation protocols, and the biomarkers used to assess oxidative status. In addition, most investigations describe associations rather than causal relationships, and only a limited number combine molecular, endocrine, physiological, and reproductive outcomes within the same experimental design. Although oxidative stress is consistently associated with impaired reproductive function in stallions, the generalizability of the available evidence remains limited. Consequently, its relative contribution compared with other biological mechanisms, as well as its interactions with endocrine, mitochondrial, inflammatory, and epigenetic pathways, remain uncertain.

Reliance on evidence from rodents, livestock, and humans remains one of the major limitations of this field. Such studies provide an essential biological framework for understanding conserved mechanisms, yet important differences in reproductive seasonality, thermoregulation, management systems, breeding intensity, and environmental exposure may substantially influence both the magnitude and biological significance of stress-induced responses in stallions. Cross-species evidence should therefore be interpreted cautiously when translating mechanistic findings into equine reproductive biology or clinical practice.

The limited number of mechanistic studies performed directly in stallions also reflects substantial practical challenges. Experimental exposure of valuable breeding stallions to environmental stress is constrained by ethical considerations, seasonal reproductive activity, and the high genetic and economic value of breeding animals. Repeated collection of testicular tissue or other invasive biological samples is rarely feasible, making direct investigation of molecular pathways particularly difficult. In addition, variation among breeds, management systems, climatic conditions, and reproductive status complicates the design of standardized field studies. Progress in this area will depend on multicentre collaborative studies, harmonized experimental protocols, and wider application of minimally invasive biomarkers integrated with physiological and reproductive assessments.

These limitations have important implications for both research and clinical interpretation. Environmental stress is likely to affect several interconnected biological pathways simultaneously, yet the relative contribution of individual mechanisms under field conditions remains unknown. Likewise, interactions among oxidative stress, endocrine regulation, mitochondrial dysfunction, inflammation, and epigenetic modifications have yet to be defined in stallions. This complexity suggests that reproductive dysfunction may occur despite apparently normal conventional semen parameters because environmental stress can impair sperm functional competence, including capacitation, acrosome reaction, and oocyte activation.

Further progress will require well-designed stallion-specific studies integrating molecular biomarkers, endocrine profiling, thermophysiological assessment, and comprehensive evaluation of sperm function. Particular priorities include identifying reliable biomarkers of early reproductive dysfunction, establishing species-specific thresholds for environmental stress, and clarifying interactions among the principal biological pathways involved in stress-induced reproductive impairment. Such integrative approaches will be essential for translating mechanistic knowledge into evidence-based reproductive management of breeding stallions.

Among environmental stressors, heat stress remains the most extensively investigated and practically relevant factor affecting stallion reproductive function. The following section therefore focuses on its effects on testicular physiology, spermatogenesis, and semen quality.

## 4 Heat stress effects on testicular function and spermatogenesis

Heat stress is a major environmental factor affecting stallion reproductive function, primarily because spermatogenesis is highly sensitive to temperature (4, 5, 19). Even small increases in testicular temperature can disrupt normal spermatogenic processes and impair semen quality, with effects that may persist long after the initial exposure. Environmental heat stress impacts stallion reproduction through several mechanisms, including direct testicular damage, oxidative stress, endocrine disruption, and impaired thermoregulation (1). These mechanisms often act simultaneously and synergistically, leading to both acute and long-term changes in reproductive function.

**Figure 2:**
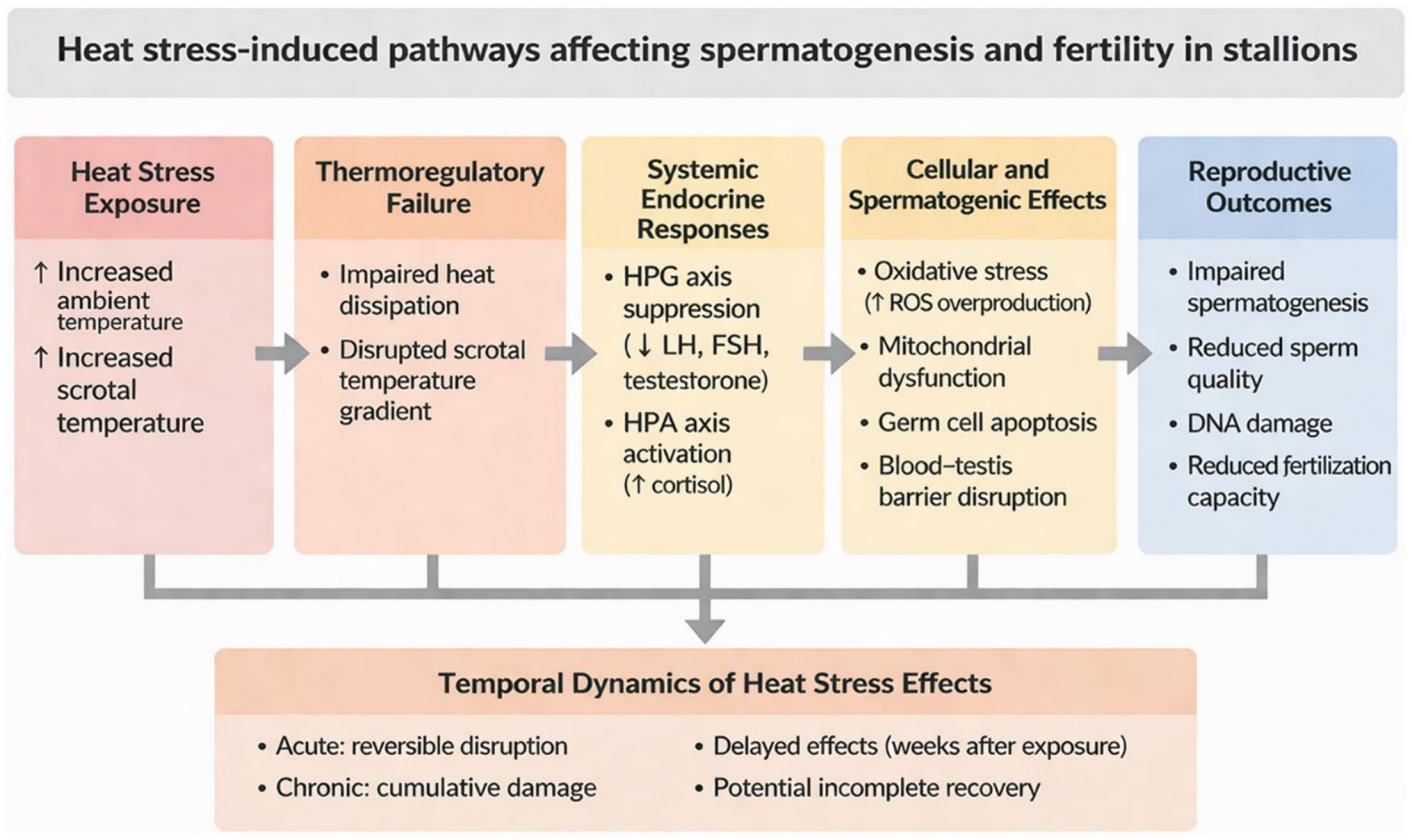
Proposed mechanisms underlying heat stress-induced impairment of spermatogenesis and fertility in stallions.

To provide a structured overview of these interconnected processes, Figure 2 summarizes the multi-level pathways linking heat stress to impaired spermatogenesis and reduced fertility in stallions.

Heat stress disrupts thermoregulation, leading to increased testicular temperature and activation of systemic endocrine responses and cellular stress pathways. These include oxidative stress, mitochondrial dysfunction, and apoptosis, which together impair testicular structure and spermatogenesis. The resulting changes in sperm quality and function contribute to reduced fertility. The figure also illustrates the temporal progression of heat stress effects from acute to chronic stages.

### 4.1 Direct heat effects on testicular tissue and structure

The seminiferous epithelium is highly sensitive to elevated temperature, and heat stress can induce structural and functional changes ranging from mild cellular disruption to complete spermatogenic arrest. Chronic scrotal heat stress has been shown to alter testicular histology, including increased interstitial cell proliferation and upregulation of intercellular adhesion molecule-1 (ICAM-1) (20). Heat stress also increases the number of macrophages (F4/80-positive cells) and fibroblasts (FSP1-positive cells), indicating activation of inflammatory pathways and tissue remodeling (20). These changes are accompanied by collagen accumulation in the interstitial tissue, suggesting progressive fibrosis, a process widely described across mammalian species under thermal stress conditions (21).

Disruption of the blood–testis barrier (BTB) is another key consequence of heat exposure. Downregulation of tight junction proteins such as zonula occludens-1 (ZO-1) allows immune cell infiltration and further germ cell damage (22). These structural alterations are closely associated with impaired spermatogenesis and reduced semen quality. Although these mechanisms are well characterized in experimental models, direct evidence in stallions remains limited. Available data are largely restricted to histological observations, with a lack of controlled heat-challenge studies in equine species, whereas strong experimental evidence from rodents, rams, and rabbits consistently demonstrates heat-induced structural damage to testicular tissue (21). Therefore, while these mechanisms are biologically plausible in stallions, their direct validation remains insufficient.

### 4.2 Spermatogenic arrest and temporal dynamics

Heat stress-induced reproductive dysfunction follows a characteristic temporal pattern. Reductions in sperm production typically appear within 1–2 weeks after exposure, with maximal impairment occurring after 4–5 weeks (6, 19). In severe cases, heat stress may result in temporary azoospermia.

Physiologically, heat stress can be classified as acute or chronic. Acute heat stress, resulting from short-term exposure, generally causes transient disruption of spermatogenesis. In contrast, chronic or repeated exposure leads to cumulative damage, delayed recovery, and an increased risk of long-term subfertility (19).

Spermatogenesis can recover after removal of the stressor; however, recovery is often delayed and may require several weeks to months, reflecting the duration of the spermatogenic cycle. Notably, semen quality tends to recover more slowly than testicular morphology (23).

Incomplete recovery has been reported, with persistent reductions in sperm concentration observed even several weeks after the stressor is removed (12). These findings suggest that repeated or severe heat stress may result in long-term or partially irreversible reproductive consequences.

Evidence describing these temporal dynamics in stallions remains moderate and is largely derived from observational data, including seasonal fertility decline and delayed recovery patterns. In contrast, controlled studies in rabbits, rodents, and rams have clearly defined the timing and progression of heat-induced spermatogenic disruption (19, 21), indicating that although general patterns are likely conserved, precise dynamics in stallions remain incompletely characterized.

### 4.3 Morphological and functional sperm alterations

Heat stress leads to the production of spermatozoa with structural and functional abnormalities, including higher proportions of morphologically abnormal sperm, reduced motility, and impaired fertilizing capacity. At the molecular level, heat stress alters gene expression in spermatogenic cells, including upregulation of stress-responsive transcripts and dysregulation of pathways involved in sperm maturation (14). Heat stress also affects calcium signaling pathways, particularly those mediated by sperm-specific calcium channels such as CatSper, which are essential for sperm hyperactivation and fertilization (24).

Oxidative stress plays a central role in mediating these alterations by promoting lipid peroxidation, protein oxidation, and DNA fragmentation, ultimately compromising sperm function (10, 11, 25). Changes in membrane composition, including alterations in cholesterol and oxysterol content, further impair membrane fluidity and capacitation processes (12).

Importantly, functional defects may occur even when sperm counts are relatively normal, highlighting that sperm quality rather than quantity is often the limiting factor in fertility under heat stress conditions (26). Functional sperm defects are relatively well documented in stallions at the phenotypic level; however, the underlying molecular mechanisms remain largely extrapolated from studies in rodents and humans, highlighting the need for species-specific investigation.

### 4.4 Endocrine and thermoregulatory responses

Heat stress also affects reproductive function through systemic endocrine pathways. Elevated temperatures disrupt the hypothalamic–pituitary–gonadal (HPG) axis, resulting in decreased secretion of luteinizing hormone and reduced testosterone production (2, 27). At the same time, activation of the hypothalamic–pituitary–adrenal (HPA) axis increases cortisol production, which further suppresses reproductive function by inhibiting gonadotropin secretion and impairing Leydig cell activity (28).

Thermoregulatory mechanisms are critical for mitigating heat stress. Stallions depend on scrotal thermoregulation and increased respiratory heat dissipation; however, these mechanisms may be inadequate during prolonged or extreme environmental conditions (29). Maintenance of testicular temperature several degrees below core body temperature is essential for normal spermatogenesis, and disruption of this gradient is a fundamental cause of heat-induced reproductive dysfunction (30).

Even brief increases in scrotal temperature can cause delayed and long-lasting effects on sperm quality. Evidence for endocrine disruption in stallions is limited, and comprehensive studies integrating hormonal, thermoregulatory, and reproductive parameters are lacking. In contrast, extensive research in other species has clearly demonstrated heat-induced endocrine alterations, supporting the plausibility of these mechanisms in stallions (19).

### 4.5 Reversibility and long-term effects

The reversibility of heat stress-induced reproductive dysfunction depends on both the intensity and duration of exposure. Short-term heat stress is generally associated with reversible changes, while chronic or repeated exposure may lead to persistent impairment of spermatogenesis (19).

Although recovery of sperm production is often observed, the threshold at which heat stress causes irreversible damage remains poorly defined, particularly in stallions. Moderate evidence suggests that seasonal heat exposure in stallions is associated with reversible changes in fertility; however, clear thresholds distinguishing reversible from irreversible damage have not been established.

In contrast, experimental studies in other species have defined temperature and duration thresholds associated with permanent reproductive impairment (21). Therefore, the long-term impact of repeated or severe heat stress in stallions remains insufficiently understood and represents a critical area for future research, especially in the context of ongoing climate change and intensification of breeding systems.

Although precise thermal thresholds for irreversible reproductive damage in stallions remain undefined, studies in other species suggest that even short-term increases of 2–3 °C above optimal testicular temperature may impair spermatogenesis, particularly when exposure is prolonged or repeated (19). These findings highlight the need to establish species-specific thermal thresholds in stallions under field conditions.

## 5 Systemic physiological responses to environmental stress

Environmental stress affects stallion reproductive function not only through direct testicular damage but also via systemic physiological pathways, particularly those involving neuroendocrine regulation and thermoregulatory adaptation (2). These systemic responses interact closely with local testicular mechanisms and may amplify or prolong the effects of environmental stress on spermatogenesis. Consequently, understanding systemic regulation is essential for interpreting the overall impact of environmental stress on stallion fertility.

### 5.1 Hypothalamic–pituitary–gonadal axis dysregulation

The hypothalamic–pituitary–gonadal (HPG) axis plays a central role in regulating male reproductive function, and its disruption represents a key pathway through which environmental stress impairs fertility. Heat stress has been shown to reduce the secretion of luteinizing hormone (LH) and follicle-stimulating hormone (FSH), both of which are essential for testosterone production and the maintenance of spermatogenesis (2, 19).

In addition, chronic heat exposure may alter steroidogenic pathways in Leydig cells, including increased aromatase activity, leading to a shift from testosterone to estradiol production and contributing to hormonal imbalance (31). Such endocrine alterations can negatively affect libido, mating behavior, and germ cell development.

At a broader mechanistic level, environmental stress may disrupt hypothalamic signaling and gonadotropin release through interactions with stress-related neuroendocrine pathways, further impairing reproductive regulation (32). These interactions highlight the close functional coupling between stress and reproductive axes.

In stallions, evidence for HPG axis dysregulation remains limited and is primarily based on observations of seasonal fluctuations in testosterone concentrations and general associations between environmental conditions and semen quality. Direct mechanistic studies linking hormonal changes to specific reproductive outcomes are scarce. In contrast, extensive experimental evidence from livestock and laboratory species clearly demonstrates heat-induced disruption of the HPG axis.

Therefore, while HPG axis dysregulation is a biologically plausible and likely important mechanism in stallions, its quantitative contribution to reproductive dysfunction remains insufficiently defined.

### 5.2 Stress hormones and the hypothalamic–pituitary–adrenal axis

Environmental stress also activates the hypothalamic–pituitary–adrenal (HPA) axis, leading to increased secretion of glucocorticoids, particularly cortisol (33). While acute activation of this pathway is adaptive, chronic elevation of glucocorticoids exerts suppressive effects on reproductive function.

Elevated cortisol levels inhibit gonadotropin release, impair Leydig cell activity, and may promote apoptosis within the testicular environment (28). Additionally, glucocorticoids interfere with kisspeptin signaling, a critical regulator of gonadotropin-releasing hormone (GnRH) secretion, further suppressing the HPG axis (34).

The inhibitory effects of glucocorticoids on reproductive function are well established across mammalian species and involve both central (hypothalamic–pituitary) and peripheral (testicular) mechanisms (32). These pathways provide a mechanistic explanation for the negative association between chronic stress and fertility.

In equine studies, hair cortisol concentration has emerged as a useful biomarker of chronic stress exposure, reflecting long-term HPA axis activity (35). Associations between cortisol levels, social hierarchy, and testosterone dynamics have been reported in horses, suggesting a link between chronic stress and reproductive physiology.

However, direct evidence linking glucocorticoid levels to semen quality and fertility outcomes in stallions remains limited. Most mechanistic insights into HPA-mediated reproductive suppression are derived from non-equine models. Thus, although the role of the HPA axis is well established biologically, its specific impact on stallion fertility requires further investigation.

### 5.3 Body temperature regulation and thermoregulatory responses

Thermoregulation is a critical determinant of reproductive function in stallions, as maintaining testicular temperature within a narrow optimal range is essential for normal spermatogenesis (19, 29). Environmental heat stress triggers physiological responses such as increased respiration rate, sweating, and peripheral vasodilation, which facilitate heat dissipation.

However, under conditions of prolonged or intense heat exposure, these compensatory mechanisms may become insufficient, resulting in elevated core and scrotal temperatures. Even transient increases in scrotal temperature can lead to delayed and long-lasting impairment of sperm quality due to the duration of the spermatogenic cycle.

Observational and experimental studies indicate that reductions in semen quality often become evident several weeks after heat exposure (5, 36). Scrotal thermography has been proposed as a non-invasive tool for assessing testicular thermal status, with studies demonstrating correlations between scrotal surface temperature and sperm abnormalities in stallions (37).

Experimental work in other species has established clear relationships between elevated testicular temperature, duration of exposure, and irreversible reproductive damage, highlighting the importance of thermal thresholds in determining reproductive outcomes (19).

In contrast, equine data are largely observational, limiting the ability to define precise thresholds at which heat exposure transitions from reversible to irreversible reproductive impairment. Therefore, while thermoregulation is fundamental to stallion fertility, the lack of species-specific quantitative data represents a major limitation for evidence-based management.

## 6 Non-thermal environmental stressors on male reproduction

Although heat stress is the most extensively studied environmental factor affecting stallion fertility, several non-thermal stressors also contribute to reproductive dysfunction through both systemic and local mechanisms. These include psychological and social stress, hypoxic exposure, environmental pollutants, and nutritional imbalance. While the biological pathways underlying these stressors—particularly endocrine disruption, oxidative stress, and mitochondrial dysfunction—are well established in other mammalian species (28, 38), equine-specific evidence remains limited and often relies on observational data or indirect inference. Consequently, the relative contribution of non-thermal stressors to stallion reproductive dysfunction remains difficult to quantify, highlighting the need for critical evaluation and targeted research.

### 6.1 Psychological and social stress

Psychological stress is an important but frequently underestimated factor influencing stallion reproductive performance. Domestic stallions are often housed under conditions that restrict social interaction, a management practice associated with increased incidence of stereotypic behaviors, aggression, and chronic stress (13).

Chronic activation of the hypothalamic–pituitary–adrenal (HPA) axis under such conditions leads to elevated glucocorticoid concentrations, which suppress gonadotropin secretion, impair Leydig cell function, and reduce testosterone production (28, 34). These endocrine pathways are well characterized across mammalian species, where chronic stress is consistently associated with impaired spermatogenesis and reduced fertility (39).

In horses, hair cortisol concentration has been proposed as a biomarker of long-term stress exposure and has been associated with variations in reproductive hormone dynamics (35). However, direct evidence linking psychological stress to semen quality and fertility outcomes in stallions remains limited, and the underlying molecular mechanisms are insufficiently defined.

Thus, while psychological stress is clearly relevant to stallion reproductive physiology, its direct impact on reproductive performance remains incompletely characterized, and causal relationships require further investigation.

### 6.2 Altitude and hypoxic stress

Hypoxic stress, such as that encountered at high altitude, represents another non-thermal environmental factor with potential reproductive consequences. Human studies have demonstrated that high-altitude exposure can significantly reduce sperm concentration and motility, with effects persisting for several weeks after return to normoxic conditions (12).

Mechanistically, hypoxia increases reactive oxygen species production, reduces antioxidant capacity, and disrupts mitochondrial function, leading to impaired spermatogenesis and altered endocrine regulation (12). In addition, hypoxia-inducible factors (HIFs) play a central role in mediating cellular adaptation to low oxygen conditions but may also interfere with normal germ cell development when chronically activated.

In stallions, direct experimental evidence is lacking, and current understanding is largely extrapolated from human and laboratory animal studies. Although these mechanisms are evolutionarily conserved, the magnitude, duration, and practical relevance of hypoxia-induced reproductive changes in stallions remain unknown.

This gap is particularly important given the increasing use of horses in high-altitude training and competition, where hypoxic stress may interact with other environmental stressors such as heat and exercise.

### 6.3 Environmental pollutants and air quality

Environmental pollutants—including particulate matter, heavy metals, pesticides, endocrine-disrupting chemicals, and microplastics—are increasingly recognized as contributors to male reproductive dysfunction across species (38, 40). These agents can impair reproductive function through oxidative stress, endocrine disruption, and direct cytotoxic effects on germ cells.

Exposure to pollutants has been associated with reduced sperm motility, increased DNA fragmentation, altered hormone profiles, and impaired fertilization capacity. Microplastics, for example, have been shown to induce oxidative damage, apoptosis, and gene expression changes in spermatozoa, highlighting their emerging relevance as reproductive toxicants (41).

Endocrine-disrupting chemicals (EDCs) may interfere with androgen signaling and steroidogenesis, further contributing to reproductive impairment (40). These mechanisms are well documented in humans and laboratory animals but remain poorly investigated in equine species.

Equine-specific data on pollutant exposure are extremely limited, and most available evidence is derived from human epidemiological studies and experimental models. Therefore, although pollutant-induced reproductive toxicity is mechanistically well supported, its real-world impact on stallion fertility remains largely speculative and represents a significant knowledge gap.

### 6.4 Nutritional and metabolic stress

Nutritional imbalance is a modifiable but often underestimated environmental factor affecting male reproductive function. Adequate intake of micronutrients such as zinc, selenium, copper, and vitamins is essential for maintaining antioxidant defenses and supporting spermatogenesis (25, 42). Deficiencies or imbalances in these nutrients increase susceptibility to oxidative stress and impair sperm quality.

The balance between omega-6 and omega-3 fatty acids plays a critical role in maintaining sperm membrane integrity and function. Diets with disproportionately high omega-6 to omega-3 ratios have been associated with reduced semen quality, impaired membrane fluidity, and altered sperm function (43).

Metabolic status, including energy balance and body condition, may further influence reproductive function through hormonal and inflammatory pathways, linking nutrition to broader systemic regulation of fertility.

In stallions, nutritional influences on reproductive function are supported mainly by observational studies linking diet composition to semen parameters. However, controlled mechanistic studies remain scarce.

Thus, while nutritional stress represents a relevant and potentially manageable factor in stallion reproduction, the specific molecular pathways involved in equine males remain insufficiently characterized, highlighting the need for targeted and controlled research.

## 7 Mitigation strategies and protective interventions

Building on the mechanistic insights and evidence gaps discussed above, mitigation strategies for environmental stress-induced reproductive dysfunction in stallions must address both cellular damage and systemic physiological dysregulation (10, 11). Environmental stress affects reproductive performance through oxidative damage, endocrine disruption, and impaired thermoregulation; therefore, effective interventions should target multiple biological levels simultaneously.

Although numerous mitigation strategies have been proposed across mammalian species, the strength of evidence supporting their application in stallions varies considerably. Most mechanistic insights derive from rodent and livestock models, whereas equine-specific validation remains limited. Consequently, distinguishing between biologically plausible approaches and those supported by direct evidence in stallions is essential for evidence-based reproductive management.

### 7.1 Antioxidant and nutraceutical approaches

Oxidative stress is a central mechanism linking environmental stress to impaired male fertility; therefore, antioxidant supplementation has been widely investigated as a potential mitigation strategy. Compounds such as melatonin, resveratrol, and other polyphenols have demonstrated the ability to reduce reactive oxygen species (ROS), stabilize mitochondrial function, and attenuate apoptosis in mammalian models (10, 11).

Melatonin has been shown to preserve Sertoli cell tight junction integrity, regulate redox balance, and protect testicular tissue under heat stress conditions (44, 45). Similarly, resveratrol has been associated with improved mitochondrial function and reduced endoplasmic reticulum stress, suggesting protective effects against heat-induced cellular damage (46).

However, in stallions, direct experimental evidence remains limited. Most equine studies report general improvements in semen parameters rather than targeted mitigation of stress-induced damage. Thus, although antioxidant strategies are mechanistically well supported, their effectiveness under field-relevant conditions in stallions remains insufficiently validated.

### 7.2 Nutritional and metabolic interventions

Nutritional strategies play an important role in maintaining reproductive function, particularly through modulation of oxidative balance and membrane stability. The ratio of omega-3 to omega-6 fatty acids is critical for sperm membrane integrity, and imbalances have been associated with reduced semen quality and impaired fertilization capacity (43).

Micronutrients such as selenium, zinc, and copper are essential for antioxidant enzyme activity and spermatogenesis (42). Deficiencies or imbalances in these nutrients increase susceptibility to oxidative damage and may exacerbate the effects of environmental stress.

In stallions, evidence for nutritional modulation of fertility is supported mainly by observational and applied studies, while mechanistic research remains limited. Therefore, although nutritional interventions are practical and widely applicable, their specific molecular effects in equine reproduction require further investigation.

### 7.3 Management and environmental modifications

Management-based strategies represent the most immediately applicable and evidence-supported approaches for mitigating environmental stress in stallions. Environmental cooling methods—including shade provision, ventilation, and water-based cooling systems—can reduce heat load and help maintain optimal testicular temperature (2, 37).

Adjustments in breeding management, such as avoiding peak heat periods and optimizing breeding schedules, may further reduce reproductive impairment (2, 5). Housing conditions also play a critical role in modulating stress responses. Stallions housed under restrictive or socially isolated conditions exhibit increased activation of the hypothalamic–pituitary–adrenal (HPA) axis, leading to elevated cortisol levels and suppression of reproductive function (13, 28, 39).

Movement and social interaction may improve metabolic resilience and reduce chronic stress responses; however, direct evidence in stallions remains limited and is largely extrapolated from studies in other species (47).

### 7.4 Assisted reproductive technologies (ART)

Assisted reproductive technologies provide additional tools for mitigating the impact of environmental stress on stallion fertility. Cryopreservation enables preservation of semen collected under optimal conditions, thereby reducing the impact of seasonal or heat-related declines in semen quality (2, 5).

Timed artificial insemination (AI) and optimized breeding strategies can improve fertilization outcomes by aligning insemination with favorable physiological conditions. Embryo transfer offers an alternative approach by bypassing compromised semen quality and ensuring successful embryo development under controlled conditions.

However, these approaches do not address the underlying mechanisms of stress-induced reproductive dysfunction and may be limited by individual variability in semen quality and cryotolerance. Therefore, ART should be considered a complementary rather than primary mitigation strategy.

### 7.5 Emerging approaches and future directions

Emerging research areas include microbiota modulation and molecular biomarker development. The gut–testis axis has been proposed as a novel pathway linking systemic metabolic status to reproductive function, with microbiota-targeted interventions shown to improve spermatogenesis and reduce oxidative stress in animal models (48).

In addition, molecular biomarkers such as sperm-borne microRNAs (miRNAs) and fertilization-related proteins, including phospholipase C zeta (PLCζ), are gaining attention as sensitive indicators of sperm functional competence and early reproductive impairment (16–18). These markers may provide earlier and more precise detection of subfertility than conventional semen analysis.

However, in stallions, these approaches remain largely unexplored, and their practical application requires further validation.

To provide a structured comparison of mitigation strategies, including their mechanisms, level of evidence, and practical applicability in stallions, the main approaches are summarized in Table 2.

**Table 2:** Mitigation strategies for environmental stress-induced reproductive dysfunction in stallions.

Intervention category	Strategy	Primary mechanism	Evidence in stallions	Evidence in other species	Practical relevance	Key references
Antioxidants	Melatonin	ROS scavenging, mitochondrial protection, anti-apoptotic signaling, BTB stabilization	Limited	Strong (rodents, livestock)	High potential; requires equine validation	(10, 44, 45)
	Resveratrol	Mitochondrial function support, ER stress reduction	Limited	Moderate	Promising but experimental	(46)
Nutritional strategies	Omega-3/omega-6 balance	Membrane stability, reduced lipid peroxidation	Limited–Moderate	Moderate	Applicable in feeding management	(43)
	Trace minerals (Se, Zn, Cu)	Antioxidant enzyme activity, spermatogenesis support	Limited	Moderate	Likely beneficial but under-studied	(42)
Management strategies	Cooling systems	Reduced thermal load, improved thermoregulation	Moderate–Strong	Strong	Highly applicable (first-line)	(2, 37)
	Breeding schedule adjustment	Avoidance of peak heat exposure	Moderate	Moderate	Directly applicable	(2, 5)
	Housing modification	Reduced HPA activation, improved welfare	Moderate	Moderate	Important but under-quantified	(13)
	Exercise/movement	Improved metabolic resilience, stress adaptation	Limited	Limited–Moderate	Potentially beneficial	(28, 39, 47)
ART	Cryopreservation	Preservation of semen during optimal periods	Strong	Strong	Highly applicable	(2, 5)
	Timed AI	Optimized fertilization window	Moderate	Moderate	Applicable	(2, 5)
	Embryo transfer	Circumvention of suboptimal semen quality	Strong	Strong	High applicability	(2)
Emerging approaches	Microbiota modulation	Gut–testis axis, inflammation control	None	Emerging (rodents)	Experimental	(48)
	Molecular biomarkers (miRNA, PLCζ)	Early detection of functional sperm defects	None	Moderate	High future potential	(16–18)

As shown in Table 2, management-based strategies currently represent the most evidence-supported and practically applicable approaches for mitigating environmental stress in stallions. In contrast, antioxidant, nutritional, and emerging molecular interventions, although biologically plausible and supported by mechanistic studies in other species, remain insufficiently validated under equine-specific conditions. This discrepancy highlights a critical gap between experimental research and field-level application and underscores the need for well-designed, stallion-specific studies to support evidence-based reproductive management. In parallel, emerging molecular biomarkers may be particularly valuable for identifying cases of subfertility in which conventional semen parameters remain normal but fertilization competence is impaired.

## 8 Conclusion and future perspectives

A central contribution of this review is the integration of current knowledge on the molecular mechanisms of environmental stress with their functional consequences for stallion fertility, emphasizing the need to move beyond conventional semen analysis toward biomarker-based and function-oriented assessment of reproductive competence.

Overall, the available evidence indicates that oxidative stress is currently the best-supported mechanism linking environmental stress with impaired reproductive function in stallions. Other mechanisms, including apoptosis, mitochondrial dysfunction, epigenetic regulation, and fertilization-related signaling, are biologically plausible but remain supported primarily by studies in other mammalian species and therefore require validation under equine-specific conditions. Consequently, current understanding of stress-induced reproductive dysfunction in stallions relies on a combination of direct equine evidence and carefully interpreted mechanistic insights from other species.

Environmental stress represents an increasingly important challenge for breeding stallions, particularly in the context of climate change, more frequent heat waves, and evolving management systems. Both thermal and non-thermal stressors may impair reproductive performance through interconnected molecular, cellular, endocrine, and physiological pathways that ultimately compromise spermatogenesis, sperm function, and fertilization potential.

From a practical perspective, management-based strategies—including optimization of housing conditions, thermal management, and breeding schedules—currently provide the strongest evidence for mitigating environmental stress in stallions. In contrast, antioxidant supplementation, nutritional interventions, molecular biomarkers, and other targeted therapeutic approaches remain promising but require further validation under equine-specific and field-relevant conditions. Assisted reproductive technologies offer valuable supportive tools but should be regarded as complementary approaches rather than direct solutions to the underlying biological mechanisms of stress-induced reproductive dysfunction. Accordingly, current management recommendations should prioritize interventions supported by direct stallion-specific evidence, whereas the molecular mechanisms summarized in this review should primarily serve as a framework for guiding future experimental research until their relative contribution has been established under equine-specific conditions.

Future research should prioritize well-designed, stallion-specific studies integrating molecular biomarkers, endocrine profiling, thermophysiological assessment, and comprehensive evaluation of sperm functional competence. Particular attention should be directed toward identifying early biomarkers of subfertility, establishing species-specific thermal thresholds, and clarifying the cumulative and potential transgenerational effects of environmental stress. Bridging the gap between mechanistic understanding and practical reproductive management will be essential for improving stallion fertility and supporting sustainable equine breeding under increasingly challenging environmental conditions.
